# An automated image-based workflow for detecting megabenthic fauna in optical images with examples from the Clarion–Clipperton Zone

**DOI:** 10.1038/s41598-023-35518-5

**Published:** 2023-05-23

**Authors:** Benson Mbani, Valentin Buck, Jens Greinert

**Affiliations:** 1 DeepSea Monitoring Group, GEOMAR Helmholtz Center for Ocean Research Kiel, Wischhofstraße 1-3, 24148 Kiel, Germany; 2 Institute of Geosciences, Kiel University, Ludewig-Meyn-Str. 10-12, 24118 Kiel, Germany

**Keywords:** Ocean sciences, Computer science

## Abstract

Recent advances in optical underwater imaging technologies enable the acquisition of huge numbers of high-resolution seafloor images during scientific expeditions. While these images contain valuable information for non-invasive monitoring of megabenthic fauna, flora and the marine ecosystem, traditional labor-intensive manual approaches for analyzing them are neither feasible nor scalable. Therefore, machine learning has been proposed as a solution, but training the respective models still requires substantial manual annotation. Here, we present an automated image-based workflow for Megabenthic Fauna Detection with Faster R-CNN (FaunD-Fast). The workflow significantly reduces the required annotation effort by automating the detection of anomalous superpixels, which are regions in underwater images that have unusual properties relative to the background seafloor. The bounding box coordinates of the detected anomalous superpixels are proposed as a set of weak annotations, which are then assigned semantic morphotype labels and used to train a Faster R-CNN object detection model. We applied this workflow to example underwater images recorded during cruise SO268 to the German and Belgian contract areas for Manganese-nodule exploration, within the Clarion–Clipperton Zone (CCZ). A performance assessment of our FaunD-Fast model showed a mean average precision of 78.1% at an intersection-over-union threshold of 0.5, which is on a par with competing models that use costly-to-acquire annotations. In more detail, the analysis of the megafauna detection results revealed that ophiuroids and xenophyophores were among the most abundant morphotypes, accounting for 62% of all the detections within the surveyed area. Investigating the regional differences between the two contract areas further revealed that both megafaunal abundance and diversity was higher in the shallower German area, which might be explainable by the higher food availability in form of sinking organic material that decreases from east-to-west across the CCZ. Since these findings are consistent with studies based on conventional image-based methods, we conclude that our automated workflow significantly reduces the required human effort, while still providing accurate estimates of megafaunal abundance and their spatial distribution. The workflow is thus useful for a quick but objective generation of baseline information to enable monitoring of remote benthic ecosystems.

## Introduction

Modern digital underwater imaging platforms such as the Ocean Floor Observation Systems (OFOS)^1^, or Automated Underwater Vehicles (AUVs)^2^ are increasingly used for the exploration and monitoring of marine seabed ecosystems by researchers, the military, as well as other stakeholders in the private sector^2^. This is because these platforms offer affordability, ease of deployment, and the ability of repeatable seafloor sampling across varying scales with high temporal and spatial resolution^3^. As a result of the recent technological developments in both hardware and software, these imaging platforms are nowadays fitted with large memory storage capabilities, as well as high-resolution photo and video camera sensors^4,5^. Consequently, camera deployments during scientific expeditions now generate huge volumes of high-resolution images of the seafloor^6^. These images carry a lot of valuable information and insights into deep sea ecosystem, such as the characteristics of seafloor substrate^7^, as well as the megabenthic fauna that inhabits these ecosystems^8^.

However, the lack of automated techniques for analyzing and interpreting these huge volumes of image datasets limits both the quality and quantity of information that can be derived from them e.g., by marine scientists focusing on deep sea geological and ecosystem monitoring^9^. Furthermore, the current manual approaches that involve the inspection and interpretation of each image by a human analyst are no longer feasible in this huge data regime, because manual annotation is expensive, subjective and thus prone to human bias^10^. Despite these challenges, underwater imaging has shown remarkable capability for documenting new discoveries in the deep ocean, using both color images and videos. In particular, the use of underwater imaging in scientific publications from domains such as marine ecological monitoring, animal behavior observation, and time-lapse imaging for temporal studies, is estimated to have increased two-fold^5^. This preference for marine imaging over traditional sampling is as a result of the ability of photographs to represent more taxa, and also because the spatial extent of the surveyed area can be determined accurately^11^. Therefore, automated workflows are needed to analyze the acquired underwater images to support these domain-specific applications. Depending on the application, these automated workflows can involve tasks such as semantic/instance segmentation, image classification, as well as object detection.

Machine learning techniques have demonstrated the potential to automate both underwater image classification and object detection tasks^12^. While image classification involves assigning a single class label to describe the content of an entire image scene (e.g. a habitat class), object detection goes further to include the identification and localization of individual instances of objects visible in the image, typically by drawing bounding boxes around them^13^. This makes object detection models particularly useful for marine scientists who aim at identifying, measuring and counting underwater objects e.g. to estimate their density and abundance^14^. While modern object detection models such as Faster R-CNN^15^ can be trained to detect objects in images with relatively high accuracy^16^, they require a lot of manually annotated bounding box coordinates along with their corresponding class labels, which is very expensive and tedious to obtain^17^. Even when expert annotators are available, the selection of example images containing megafauna to be presented to the annotators can be very challenging; this is more pronounced in underwater image datasets of deep seabed areas because the frequency and diversity of megafauna is very low at greater depths, which implies that only a small proportion of the underwater image dataset contain visible megafauna^18^. In OFOS/AUV deployments where tens to hundreds of thousands of images have been recorded, the task of selecting example images with visible megafauna does pose a serious challenge.

A proposed workflow for automated detection of megabenthic fauna should therefore incorporate (semi) automated ways of reducing and/or complementing the effort of human annotators e.g. by efficiently expediting the generation of annotations from the optical underwater images^19,20^. This automation should facilitate both the selection of example images with visible megafauna to be presented to the annotators, as well as the generation of a set of weak annotations to be refined later. In this context, weak annotations are imprecise or noisy annotations that can be obtained cheaply using unsupervised approaches^21^. An example of a set of weak annotations would be bounding box coordinates that only partly cover the body of an ophiuroid (e.g., its central disk) while leaving out its arms. When available, these weak annotations greatly reduce the effort required from expert annotators, since their tasks are essentially reduced to: (a) refining the provided bounding box coordinates to precisely cover the entire megabenthic fauna; (b) annotating additional megabenthic fauna that are not part of the provided weak annotations; and (c) assigning the correct morphotype class labels^17^. One computationally cheap way of generating these weak annotations is through the analysis of image superpixels^22^.

Superpixels are partitions of an image where each partition comprises a group of pixels with similar perceptual characteristics^23^. In underwater images recorded from a relatively homogenous seabed substrate e.g. sandy or muddy bottoms in the deep sea, these superpixels generally correspond to the objects occurring on the seafloor, such as megabenthic fauna, rocks or marine litter^24^. Since the frequency of megabenthic fauna on the deep seafloor is very low compared to background objects such as the soft sediment or rock debris^18^, those superpixels that correspond to megabenthic fauna can be considered anomalous. This is because their visual properties are clearly different to the background seafloor^25^. However, in order to automatically distinguish between normal and anomalous superpixels, their visual properties must first be extracted and encoded into feature vectors. Although this can be achieved by manually identifying the distinguishing characteristics of the superpixels (e.g. color and texture), this process requires significant amount of domain expertise and experience to be done correctly^6^. An alternative approach is to automatically learn these properties directly from the superpixels e.g., by using convolutional variational autoencoders for feature extraction^26^. Anomaly detection algorithms such as iForest^27^ can then be applied to these features, so that anomalous superpixels can be detected and presented to expert annotators as weak annotations for refinement, labeling, and subsequent training of an object detection model e.g., Faster R-CNN^15^.

Past studies have proposed various approaches for seafloor substrate classification^28,29^, and in particular for underwater object detection. Traditional image processing techniques have been used to estimate the coverage of seagrass meadows in Croatia through classification of irregular image segments^30^, as well as in the Palma bay using regular square image tiles^31^. In the same direction, a saliency-based workflow was implemented to approximate background regions of the image to detect underwater ‘foreground’ objects^32^, whereas contrast stretching and adaptive thresholding has been used to segment and subsequently detect underwater objects^33^. Further, a combination of Laplacian filtering, histogram equalization and blob detection has also been used to detect underwater objects^34^. Regarding the detection of objects and human artifacts on the seafloor, a region-based approach was used to detect marine litter in Greek waters^24^, whereas geometric reasoning was employed for the detection of pipelines on the seabed^35^. In another study^36^, underwater robots were used to perform color restoration in real time in order to improve accuracy when detecting and tracking mobile objects, whereas template matching was used to detect and track objects from images recorded using an underwater robot platform^37^. By modeling the propagation of light through the water column, a workflow was implemented to detect underwater objects using monocular vision^38^, while another one was proposed to detect underwater objects by leveraging collimated regions of artificial lighting^39^. Most recent studies employ deep learning approaches: an architecture was proposed for detecting objects in complex underwater imaging environments based on feature enhancements and anchor refinement^40^, whereas an augmentation strategy was used to simulate e.g. overlaps and occlusions to improve underwater object detection accuracy^41^. Similarly, an architecture was proposed to detect underwater objects by accounting for underwater image degradation through the joint learning of color conversion and object detection^42^. Finally, a variational autoencoder architecture was used to distinguish salient regions from the background based on reconstruction residuals^25^.

**Figure 1:**
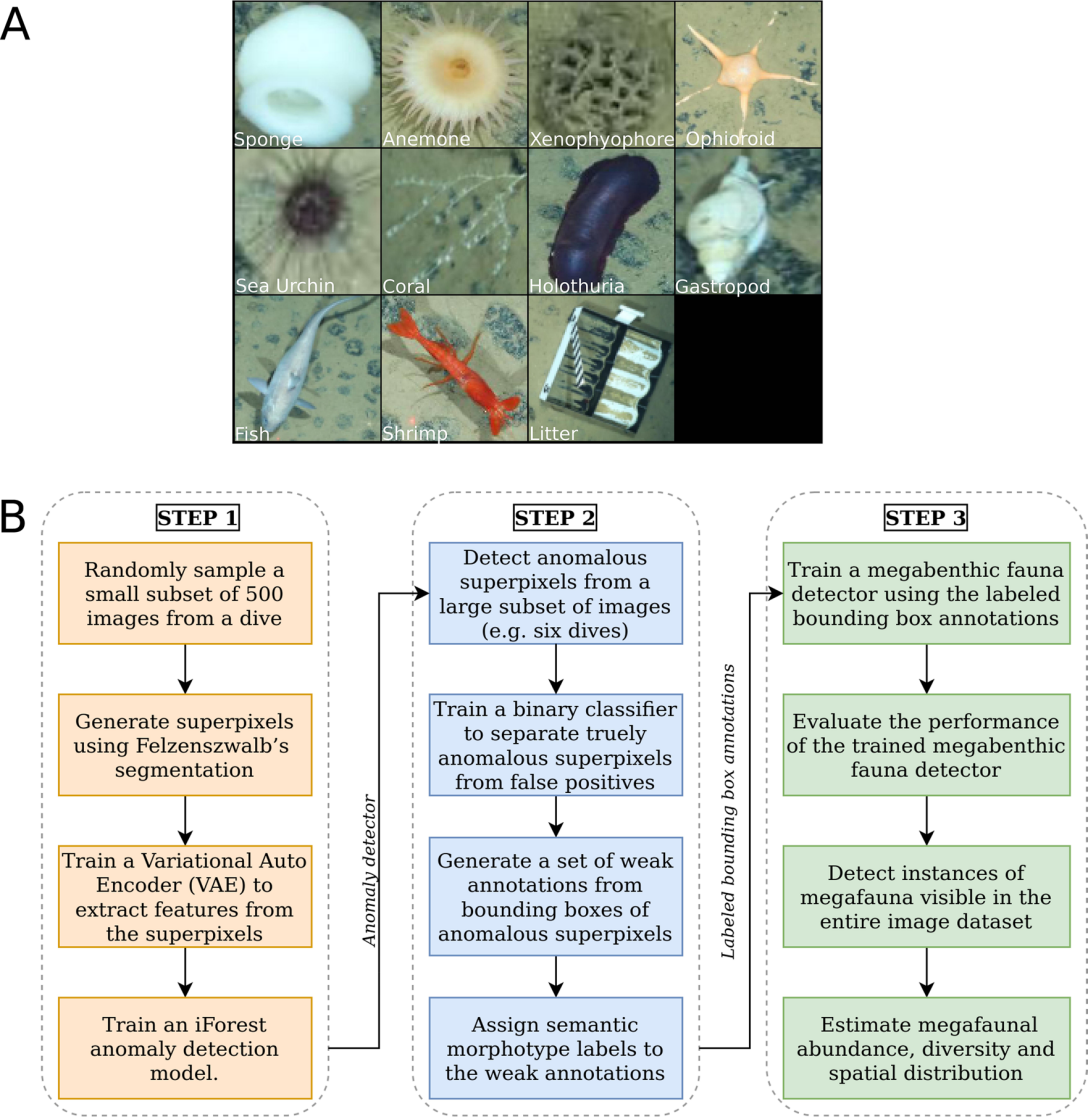
Overview of our optical image-based megabenthic fauna detection framework. (**A**) Examples of target morphotypes, including litter, that were detected on the seafloor. (**B**) Schematic diagram of our three-step workflow: The first step (automatically) generates superpixels from a small subset of sampled images, and (automatically) extracts their features for training an anomaly detection model. The second step detects anomalous superpixels (automatically) from a larger subset of images, and (semi-automatically) proposes them as weak annotations ready to be post-processed and assigned semantic morphotype labels (manually). The final step uses the semantic annotations to (automatically) train a Faster R-CNN object detection model, which then detects instances of benthic megafauna visible in the entire underwater image dataset (automatically), allowing for the estimation of megafaunal abundance, diversity and spatial distribution (manually).

In this study, we propose a three-stage workflow for automatically detecting megabenthic fauna from optical underwater images; examples of target megabenthic fauna classes (morphotypes) for this study are shown in Fig. 1A, whereas the proposed workflow is conceptualized schematically in Fig. 1B.

The first stage involves generating superpixels from a small subset comprising e.g., 500 images per dive/camera tow, which are randomly sampled to reduce computational cost in this stage. A variational autoencoder is then applied to these superpixels to extract feature vectors, which are used to train an iForest anomaly detection model. The second stage applies the trained iForest model dive-by-dive to detect anomalous superpixels from a much larger subset of underwater images e.g., comprising six out of twelve dives. A binary classifier is used for post-processing the anomalous detections to remove false positives. The bounding boxes of the truly anomalous superpixels are then presented as a set of weak annotations to an expert annotator, who assigns semantic morphotype labels to them. The final stage uses the semantic annotations to train and evaluate a Faster R-CNN object detection model, which is subsequently used to detect and classify megafauna visible in all images from all dives. These georeferenced detections are finally used to estimate abundance, diversity and spatial distribution of megabenthic fauna within the working area.

Our approach significantly reduces the required human annotation effort, since the user input is only required to post-process the automatically generated weak annotations, and assign them semantic labels. Furthermore, we have also open sourced the python scripts implementing each component of the above-described workflow, along with detailed documentation to guide users to get started using and/or extending our workflow. Thus, our approach offers a convenient underwater image annotation solution for the marine imaging community, allowing them to quickly generate accurate baseline information that allows for efficient and repeatable characterization of ecological and spatial distribution of remote marine benthic communities, including their habitats, at varying spatio-temporal scales.

## Results

### Visualization of superpixel separation

This section provides projections of both normal and anomalous superpixels onto a two-dimensional feature space for visualization purposes. These projections are obtained by applying Principal Components Analysis (PCA) onto the data matrix of feature vectors extracted from the superpixels. A grid view of truly anomalous superpixels is also provided.

#### Superpixels for training the iForest anomaly detector

**Figure 2:**
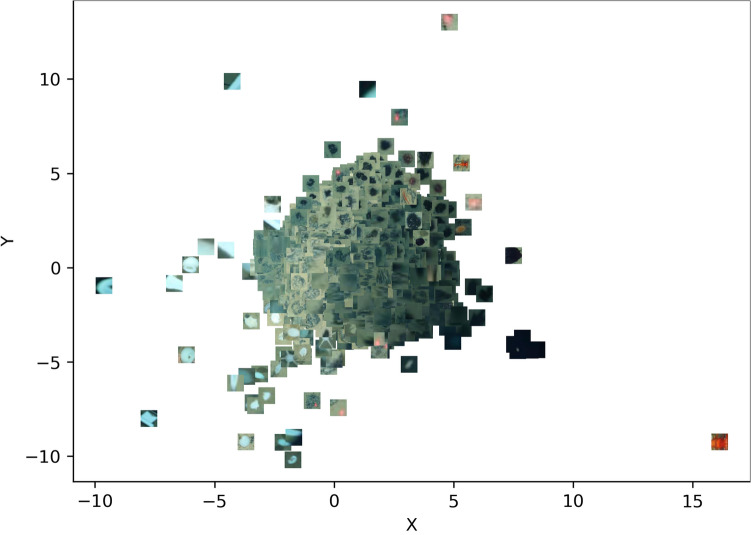
Feature space projection of superpixels whose features were used to train the anomaly detection model. Those representing the background seafloor are densely distributed around the origin of the feature space, whereas few anomalous superpixels are sparsely distributed further away towards the periphery of the feature space.

Figure 2 shows the feature space representation of superpixels used to train the iForest model. The figure clearly shows that superpixels representing the background seafloor are densely distributed around the center of the feature space since they are visually similar, while those with unusual visual characteristics are distributed farther away towards the periphery of the feature space. Therefore, the background seafloor superpixels are obviously the majority, and were considered the ‘normal’ in this study.

#### Detected anomalous superpixels

**Figure 3:**
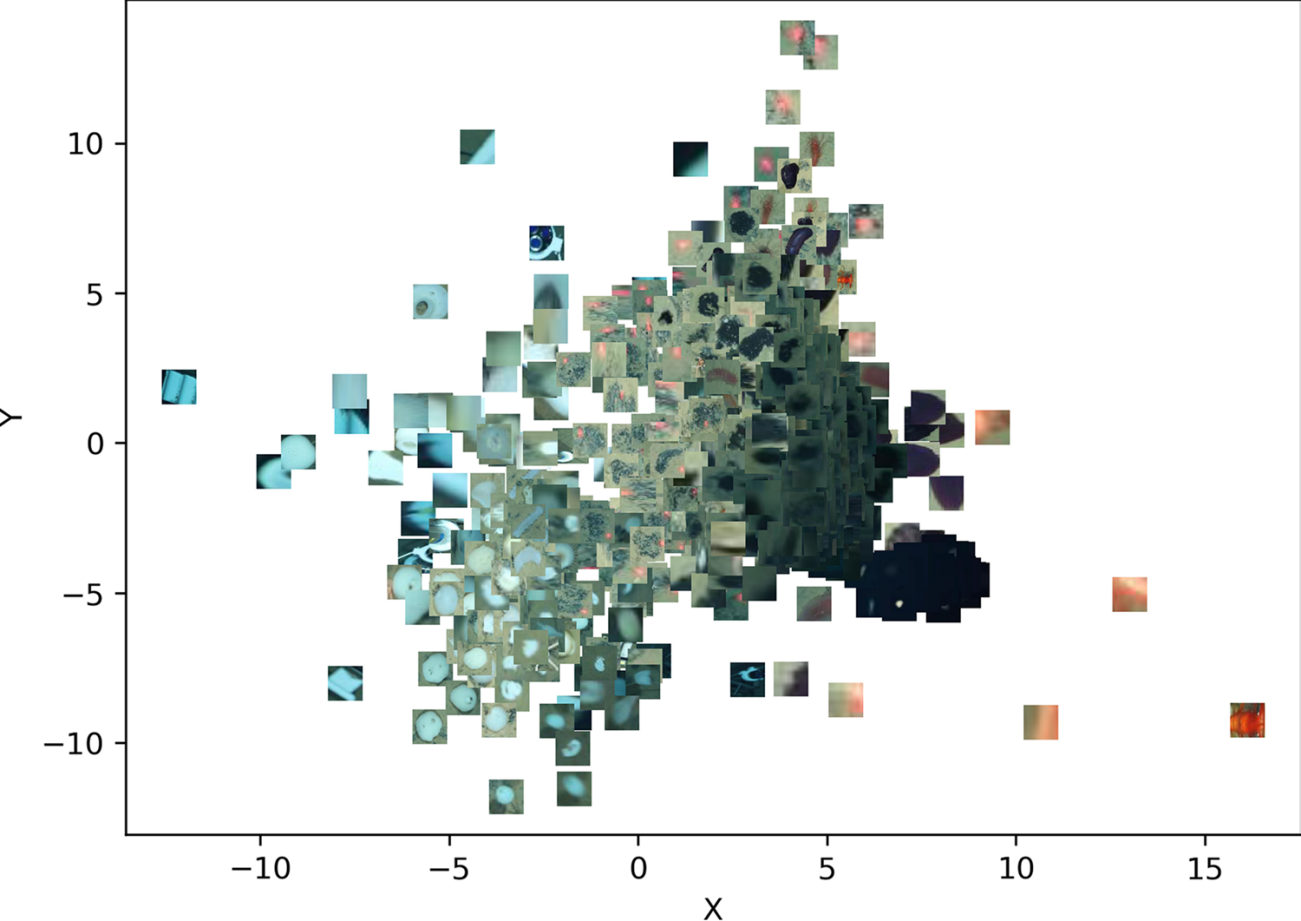
Feature space projection of the anomalous superpixels detected from images in dive 126. While some false positives such as red lasers and dark pixels of the water column were also detected, the rest of the anomalous detections represent potential instances of megafauna whose bounding boxes can be proposed as a set of weak annotations.

Figure 3 shows the feature space representation of the anomalous superpixels that were detected from dive 126. While some of the detected anomalies are false positives e.g., the red laser points and unusually dark objects on the seabed, the rest of the detected anomalies indeed represent interesting objects e.g., megabenthic fauna, or other unusual objects worth investigating.

#### Weak annotations (truly anomalous superpixels)

As mentioned above, some of the detected anomalous superpixels are false positives that do not represent megabenthic fauna. Thus, it was necessary to remove these false positives, and retain only the truly anomalous superpixels during further processing. Below, we provide the results of two post-processing strategies that we attempted: setting a threshold on the anomaly score; and training a supervised binary classifier.

**Figure 4:**
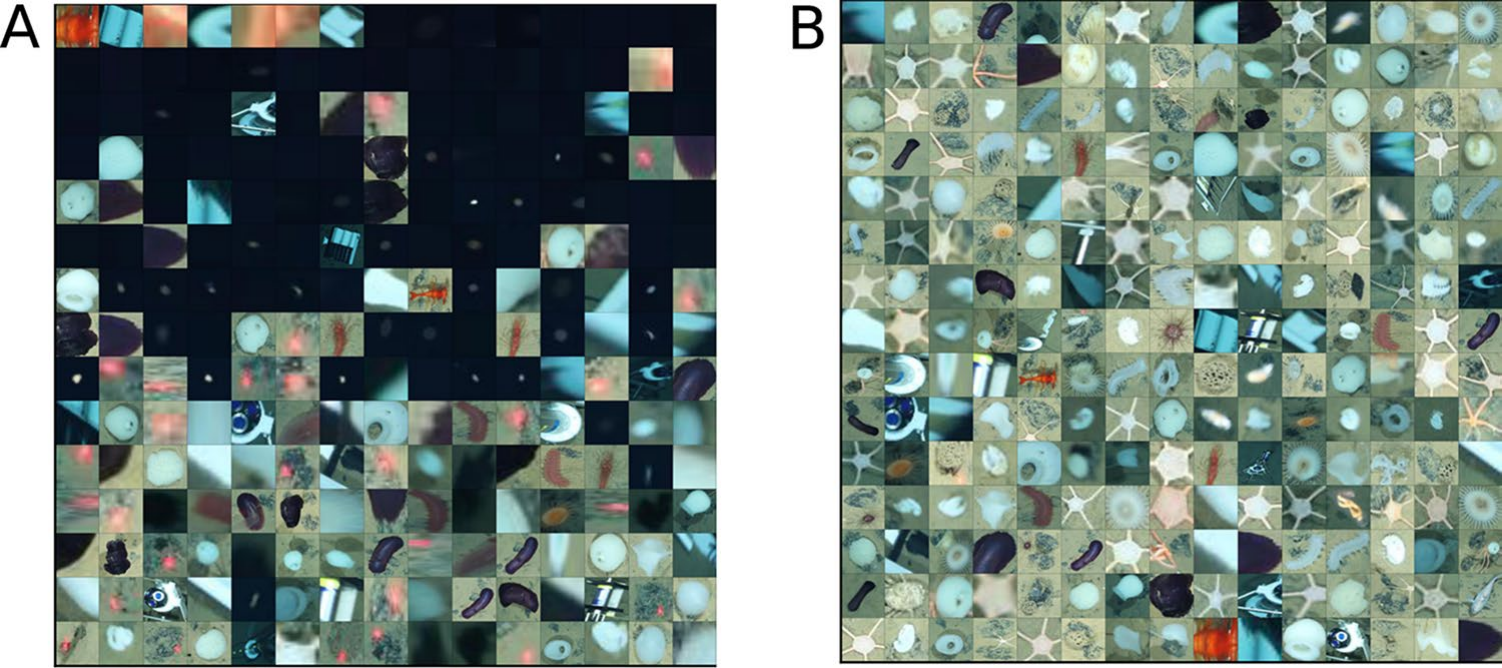
Grids of image patches showing truly anomalous superpixels obtained by (**A**) Thresholding the anomaly scores, and (**B**) Binary classifier trained with examples of both true and false positives. Thresholding produces undesired results e.g., the red laser points and the dark patches from the water column. On the other hand, the binary classifier results in a set of truly anomalous superpixels that are clearly instances of megabenthic fauna. These were proposed as weak annotations.

Figure 4A shows the results of post processing obtained by setting a 75th percentile threshold on the anomaly scores assigned to the anomalous superpixels; superpixels with anomaly scores greater than the set threshold were marked as truly anomalous. While the superpixels are visually anomalous in some way, some of them still represent objects that are not of interest in this study e.g., the red laser points, and white spots surrounded by black pixels. Because of this, we concluded that thresholding based on anomalous scores alone was not sufficient to distinguish truly anomalous superpixels from false positives. There was also no obvious way of determining the suitable anomaly score threshold.

Figure 4B shows the truly anomalous superpixels that were obtained by using our supervised binary classifier. Unlike the thresholding approach, the binary classifier correctly identified the set of truly anomalous superpixels. Bounding box coordinates of these truly anomalous superpixels were then proposed as a set of weak annotations.

### Training and evaluating FaunD-Fast model

The weak annotations still lack semantic morphotype labels, and are therefore not directly usable. In this section, we provide the results of the semantic labeling exercise involving an expert annotator, as well as the results of the performance evaluation of the Faster R-CNN object detection model that was trained using these annotations.

#### Semantic labeling of the weak annotations

A human expert manually inspected all the weak annotations and assigned them semantic morphotype labels. The expert also annotated instances of megafauna that were visible in the images, but missing from the weak annotations. This semantic labeling exercise was repeated twice (after shuffling the weak annotations) to reduce biases e.g., due to human fatigue.

Supplementary Figure S1 shows a screenshot of our superpixel annotation software during an active semantic labeling session. The left panel of the software shows all truly anomalous superpixels, whereas the right panel displays their bounding box extents overlaid on the respective parent images. The bottom panel shows the morphotypes that were considered in this study. These include: anemone, coral, fish, gastropod, holothurian, ophiuroid, sea urchin, shrimp, sponge and xenophyophore.

**Figure 5:**
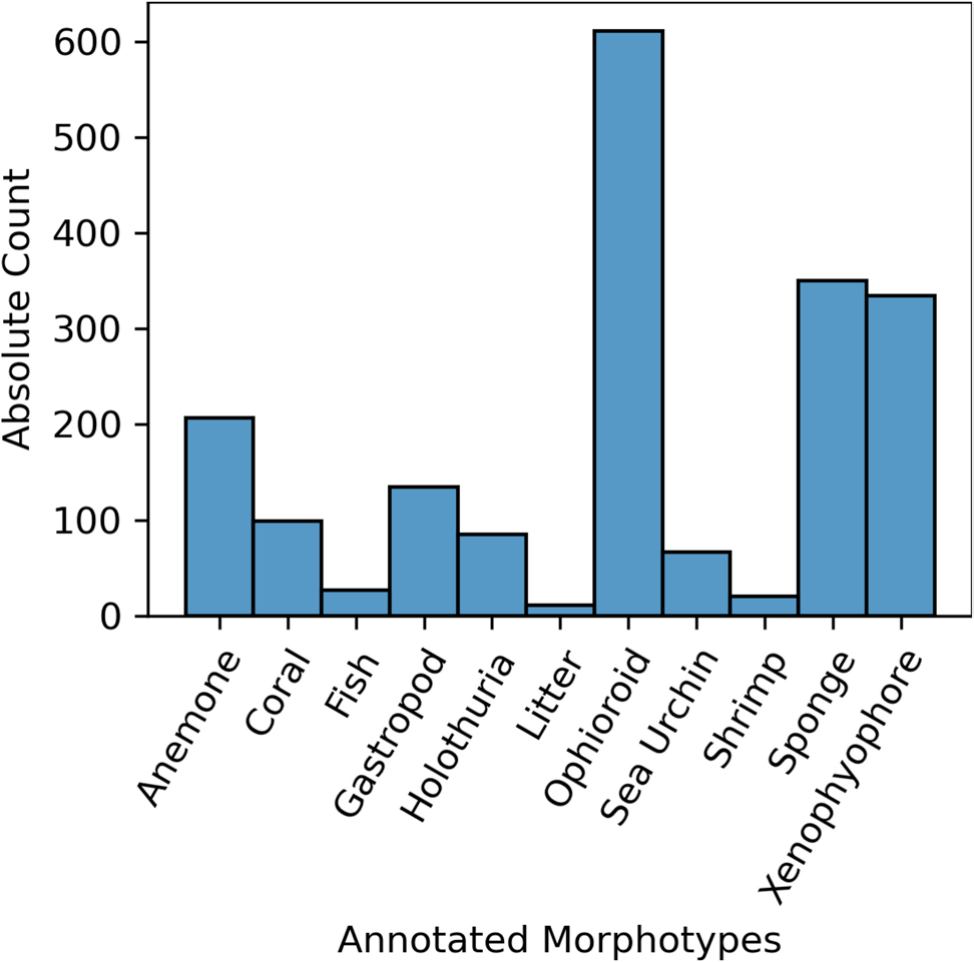
Distribution of the annotated morphotypes after exporting from the annotation software. Ophiuroids, sponges and xenophyophores were among the dominant morphotypes in the annotated dataset.

Figure 5 shows the distribution of the annotated morphotypes. In terms of proportions, the dominant morphotypes were ophiuroids (31%), sponges (18%), xenophyophores (17%) and anemones (11%). The other morphotypes had occurrences of less than 10%. These are the annotations we used to train and evaluate our FaunD-Fast model; we have provided these annotations as a csv file in supplementary Table S1.

#### Performance evaluation of FaunD-Fast model

Our FaunD-Fast model achieved an average precision (*AP*_.50_) score of 78.1% at an IoU threshold of 0.5. The model performance was higher when detecting large-sized objects/megafauna, as can be shown by the values of (*AP*_*large*_) and (*AR*_*large*_) metric categories that are both greater or equal to 70% (see Table 1). On the other hand, the model’s performance was lower when detecting small-sized objects, since both their average precision (*AP*_*small*_) and recall (*AR*_*small*_) values were less than 20%.

**Table 1:** Performance comparison relative to other state-of-the art benthic fauna detection models^43^.

Model	*AP*_.50:.95_	*AP*_.50_	*AP*_*small*_	*AP*_*medium*_	*AP*_*large*_	*AR*_1_	*AR*_10_	*AR*_100_	*AR*_*small*_	*AR*_*medium*_	*AR*_*large*_
FaunD-Fast (Ours)	46.5	**78.1**	12.7	42.0	**69.7**	**50.0**	52.0	52.4	16.2	50.0	73.2
CM-X-101/Baseline	41.7	68.2	25.3	29.3	54.7	21.6	51.6	55.2	25.4	45.1	70.8
CM-X-101*/*Synth	48.8	71.0	27.4	39.1	62.8	24.7	58.8	**64.2**	27.9	**57.3**	77.1
CM-X-101*/*Synth-Blcd	**51.8**	76.7	27.5	40.2	66.1	25.7	**59.0**	63.9	27.9	55.7	**77.9**
CM-X-101*/*Trad. Augm	48.8	75.0	26.9	38.6	58.5	23.0	55.3	58.9	27.2	50.1	72.6
CM-X-101*/*Fusion	51.7	74.1	27.1	**42.1**	65.1	24.9	57.6	61.6	27.5	52.2	77.6
CM-V-99*/*Synth	47.9	72.0	**27.9**	37.0	62.8	23.6	56.6	61.9	28.3	52.6	77.1
CM-L-M*/*Synth	27.3	48.6	19.1	19.0	40.0	18.3	39.1	43.7	20.0	34.4	59.5
M-X-101*/*Synth	33.3	53.2	13.2	22.7	53.0	20.7	39.2	40.0	13.2	30.6	60.7
R-X-101*/*Synth	47.8	70.7	**27.9**	37.1	62.2	24.2	56.6	61.9	**28.4**	53.8	76.7

The highest scores per metric category are indicated in bold.

When compared to competing state-of-the art models from the empirical evaluation in Lütjens et al^43^, their best model (*CM-X-101/Synth-Blcd)* performed better than ours with regards to the (*AP*_.50:0.95_) metric category, which is obtained by averaging the precision values over multiple IoU thresholds. In contrast, our model performed better than all the compared models with regards to the (*AP*_.50_), which is the precision at a single (absolute) IoU threshold of 0.5. In addition, our model also performed better than the others with regards to the (*AP*_*large*_) and (*AR*_1_) metric categories; this implies that our model was good at detecting large-sized megafauna. However, all the compared models reported very low precision and recall scores when detecting small-sized objects.

**Figure 6:**
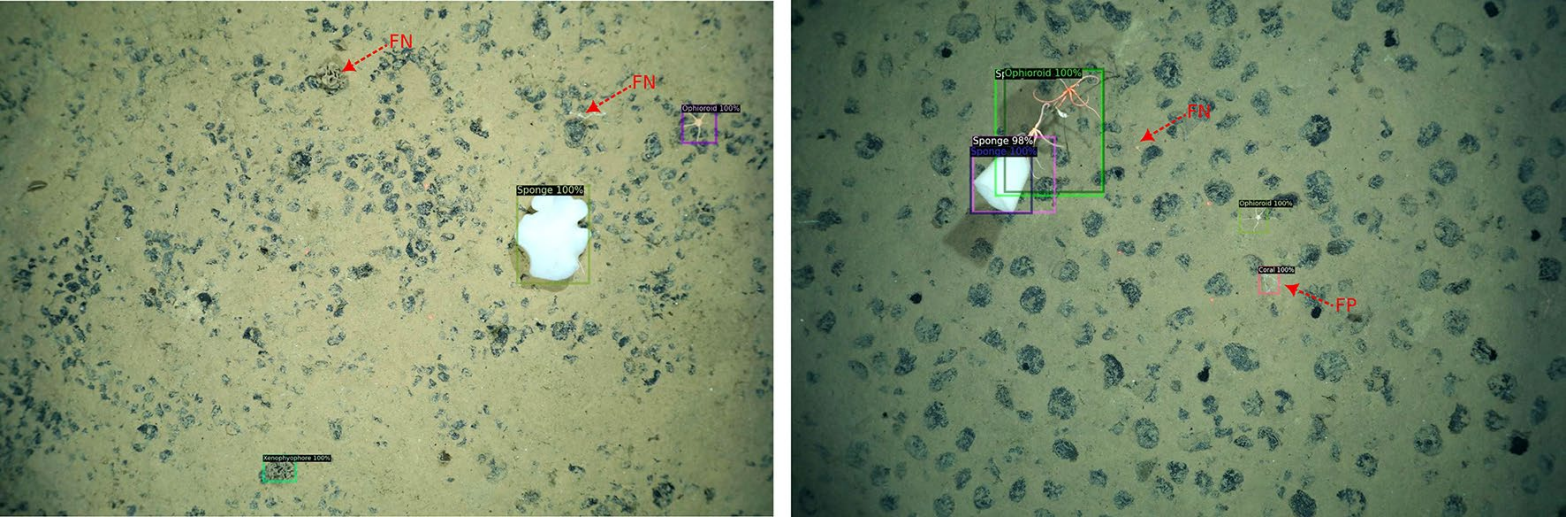
Examples images showing correctly detected instances of megabenthic fauna, as well as instances of both false positives (FP) and false negatives (FN). Morphotypes whose visual characteristics is similar to the seafloor substrate (e.g. xenophyophores and partially burrowed ophioroids) resulted in a higher proportion of false negatives. Also, incorrect detection/localization was observed in instances where morphotypes formed associations with each other e.g. between ophiuroids and sponges.

To show our model’s performance on the semantic morphotype classes, we present the confusion matrix in Supplementary Figure S2. The confusion matrix shows that majority of the morphotypes were correctly localized and identified. In particular, xenophyophores and ophiuroids contributed towards the largest proportion of false negatives. This could be because the visual characteristics of some xenophyophores and partially burrowed ophiuroids are similar to the seabed substrate, which makes them difficult to detect. In addition to this, Fig. 6 shows that in instances characterized by associations among morphotypes e.g., between ophiuroids and sponges/corals, the model made incorrect or low-confidence predictions.

Although none of the compared models (in Table 1) was able to achieve the highest score across all the metric categories, these quantitative evaluation results show that overall, the performance of our model was on a par with the best performing state-of-the art alternative(s), yet our approach required less manual annotation effort.

### Abundance, diversity and spatial distribution of the detected megabenthic fauna

**Figure 7:**
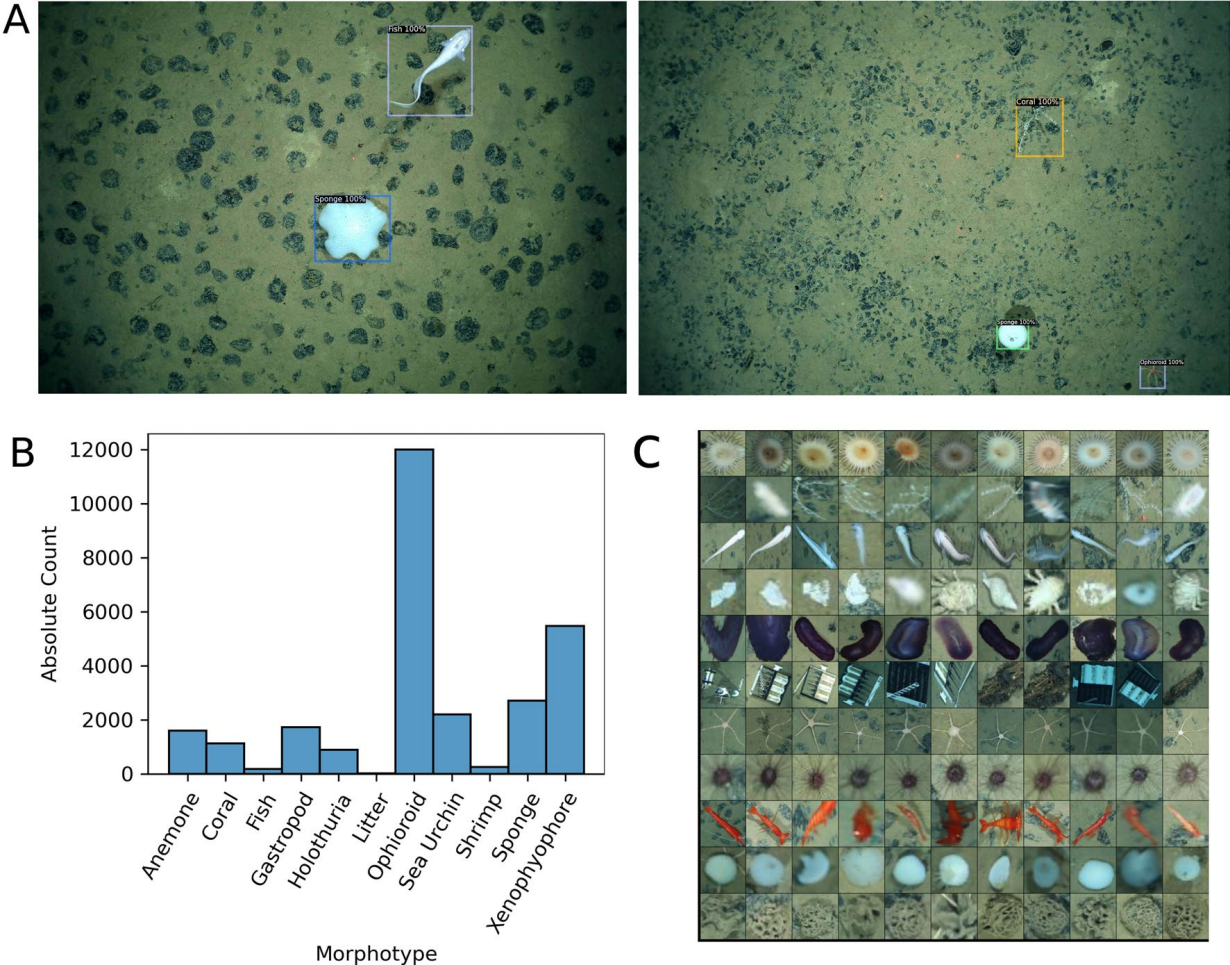
(**A**) Qualitative examples of detected instances of megabenthic fauna (**B**) Distribution of morphotypes that were detected by our FaunD-Fast model. This distribution is similar in shape to that of annotations (see Fig. 5), except the FaunD-Fast detected a lot more instances of megafauna. (**C**) Grid view showing megafauna examples grouped by morphotypes in every row of the grid; the morphotype label for each row follows the same order as in panel (**B**).

Figure 7A shows qualitative examples of correctly identified and localized megafauna as detected by our FaunD-Fast model. In total, 27,954 individual instances of megabenthic fauna were detected from the entire image dataset. Furthermore, we estimated the megafaunal abundance within German area to be approximately 0.247 ind. m^−2^ while in the Belgian area it was approximately 0.200 ind. m^−2^.

Figure 7B shows the distribution of the detected morphotypes. Ophiuroids and xenophyophores were the most dominant morphotypes accounting for 62% of all the detections. Other species are sponges (9.6%), sea urchins (7.8%), gastropods (6.1%), anemones (5.7%), corals (4.0%) and holothurians (3.1%). The rest such as fish and shrimp have occurrences of less than 1%. Apart from ophiuroids which are abundant in both contract areas, the German seabed is predominantly occupied by xenophyophores (22.8%) and sponges (10%); the Belgian contract area was predominantly occupied by sea urchins (34.9%), anemones (14.5%) and sponges (10%).

Figure 7C shows few examples of detected morphotypes, while a table summarizing all the detections is provided in the Supplementary Table S2.

**Figure 8:**
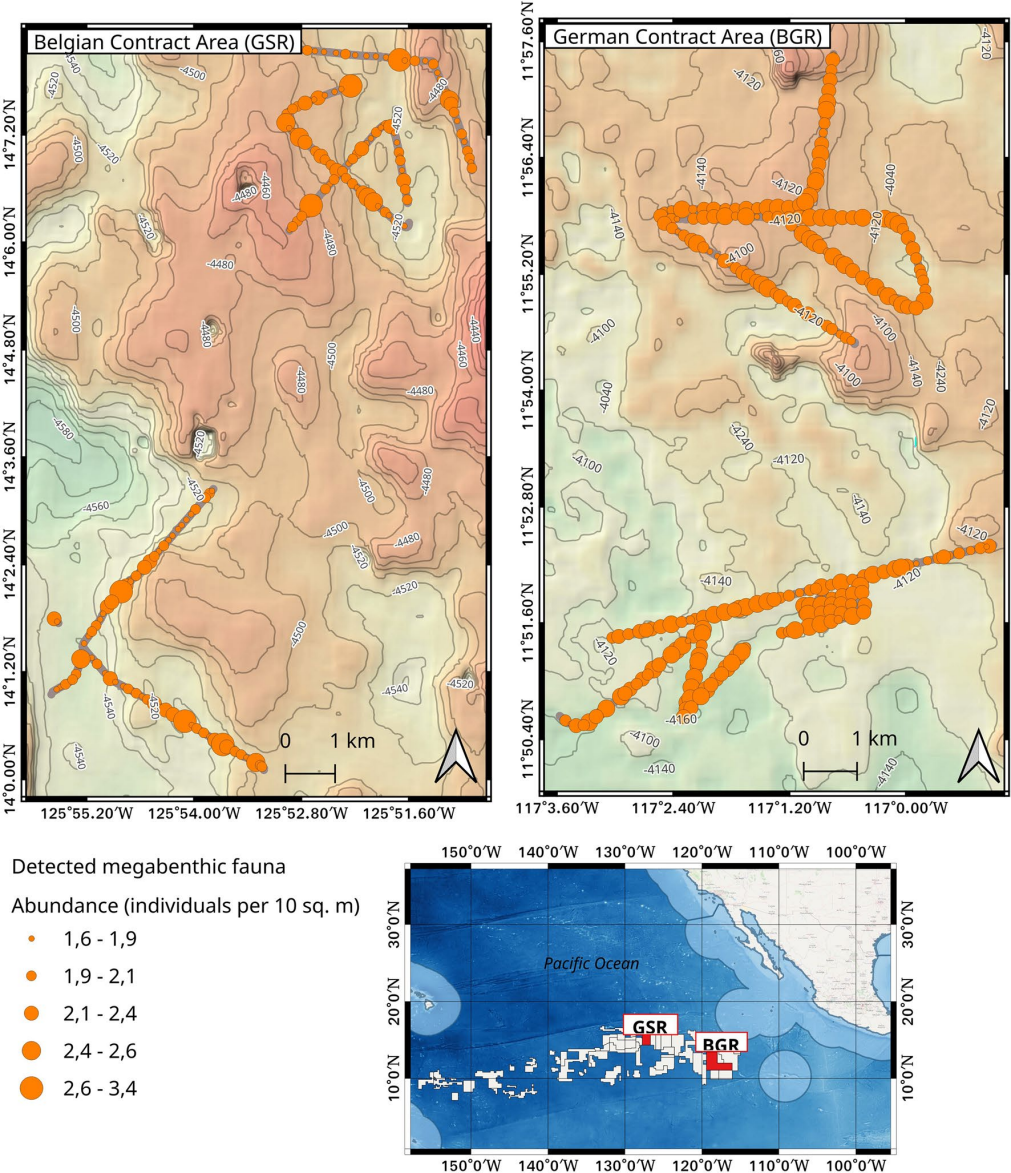
Map view showing spatial distribution of detected megabenthic fauna along camera deployment tracks in both the German and Belgian contract areas. The German seabed contained higher abundance of megafauna, probably because of availability of food in form of sinking organic material since it is on average shallower than the Belgian seabed. The map was generated using the open source QGIS software v3.2 (https://www.qgis.org/).

The spatial distribution of the detected megabenthic fauna is shown in Fig. 8. The map shows that the German contract area contains a higher abundance of megafauna compared to the Belgian area (see details in the “Discussion” section below). For ease of visualization, the detections (points) were spatially clustered by first gridding them into square blocks of size 200 m, and then normalizing the absolute count of megafauna based on the visual footprint of each respective block (in square meters). Thus, the symbology size is proportional to the abundance of megafauna within each spatial cluster/block.

## Discussion

The proposed megabenthic fauna detection workflow comprised the generation of weak annotations from superpixels, semantic morphotype labeling of the proposed weak annotations, and finally the usage of these annotations to train our FaunD-Fast model. Below, we discuss key aspects of these proposed workflow steps, and provide a more detailed discussion of the spatial distribution, density and diversity of the detected megabenthic fauna. We also suggest a few recommendations for further research.

The hyperparameter settings of the segmentation algorithm control the geometrical properties of the generated superpixels e.g., shape (regular or irregular), and size (large or small). Given that the used seafloor images comprised background seabed substrate (Mn-nodules) and other objects of varying shapes and sizes, we had to manually determine the optimal values of hyperparameters such as scale (pixel size) and width of the gaussian filter that smooths the image prior to segmentation. These parameters must be properly tuned if the workflow is applied to other underwater image datasets. If this is not done thoroughly, the generated superpixels may be of low quality hence negatively affecting the accuracy of downstream analysis. In our case, we observed that a poor choice of these hyperparameters led to inaccurate segmentation of certain morphotypes of interest, especially those with extended arms and spikes e.g., ophiuroids and sea urchins. In a related previous study using a fish dataset^44^, the authors also emphasize that segmentation hyperparameters must be properly optimized before being applied to underwater images recorded from challenging environments e.g. where both illumination conditions and background seafloor properties vary within and between datasets. In addition to the hyperparameter settings, we also had to select a subset of images whose superpixels would be used to train the iForest anomaly detection model; our subset comprised 500 randomly sampled images that generated 125,000 superpixels. We chose this sample size because it fit into our CPU memory at train time (a larger subset should be used if more memory and compute is available e.g., in HPCs). In any case, we observed that this random sampling approach potentially resulted in a more representative subset compared to e.g., a manual sampling approach. This is because the huge volume of images could easily cause the analyst to mistakenly choose a subset of images that represent more or less the same region of the seafloor (consecutive images were recorded every 10 s, and are stored on disk in order of their acquisition time), or those which look ‘interesting’.

The majority of the superpixels generated for training the anomaly detection model represented background seafloor, compared to the relatively few megafauna superpixels (see Fig. 2). This observation was expected since the abundance of megabenthic fauna in the deep ocean is typically very low, due to the low organic carbon flux/little food availability in great water depth^18^. Similar findings have been reported in studies that examined the relationship between megabenthic fauna communities and bathymetric gradients e.g.^45–48^. As a result, we observed that the detection of anomalous superpixels was relatively straightforward: they are visually rather different from the background, and are thus distributed further from the center of the feature space where the majority of the background superpixels clustered (see Fig. 3). The approach of analyzing the visual properties of superpixels has been employed in previous studies aimed at identifying the boundaries of interesting objects on seafloor images^32,44^, as well as on terrestrial images^25^. In contrast to these three publications, our approach is different because we do not make assumptions regarding the region of the image in which the foreground objects are expected e.g., in the central portion of the image. Instead, we assume that our objects of interest will be located anywhere on the image, and thus our trained iForest model detects anomalous superpixels based purely on features extracted from the superpixels. The detected anomalies still had to be post-processed to remove false positives, which occurred because we intentionally set iForest’s ‘contamination factor’ setting to a high value (0.4); this caused it to flag as many anomalous superpixels as possible (both obvious and subtle). We did this because the visual properties of some megafauna of interest such as xenophyophores are very similar to background seafloor substrate, yet we wanted the iForest model to detect them as well. In a previous study on image-based megafauna community assessment in the DISCOL area of the south Pacific Ocean^49^, the authors also point out the difficulty that even expert human annotators face when it comes to distinguishing certain morphotypes from background seafloor.

The bounding box coordinates of the detected anomalous superpixels were proposed as a set of weak annotations to be inspected and labeled by an expert annotator. This semi-automated approach significantly reduced the human effort required in generating training annotations, because it was no longer necessary for the expert annotator to manually inspect a large number of images with the aim of identifying the few that contain visible fauna, and then mark these fauna manually. The available bounding box further reduced the work of the expert annotators to just verifying and assigning semantic morphotype labels. Since these annotations were generated from unsupervised segmentation and anomaly detection methods, they are not sufficient on their own (in quantity and quality) to estimate the abundance of megafauna on the seabed for the entire dataset. They just represent training examples for a state-of-the art object detection model, which can then be applied to the entire image dataset. After using the generated annotations to train our FaunD-Fast model, we achieved a good performance (78.1%) that is on a par with other state-of-the art object detection models, which were trained in previous studies^43^ using underwater image dataset comparable to ours (see Table 1). Given that our FaunD-Fast model achieves this good performance for a fraction of the annotation effort implies that it is scalable to other applications involving huge volumes of underwater imagery. Another observation is that since our model uses the two-stage Faster R-CNN architecture that prioritizes prediction accuracy over speed^15^, the results of our comparison with other state-of-the art models (in Table 1) shows that our approach is suitable for deployment on workstations with good processing capability e.g. GPU and memory resources. For faster detection on edge devices and smaller computers, the comparison implies that a single stage detector is probably more suitable; future research could explore this further. Also, none of the compared state-of-the-art models achieved the highest score across all the metrics, which could be because each model are trained to optimize a different loss function^50^. Finally, the comparison revealed that the prediction accuracy for small-sized megafauna was consistently lower than for large-sized megafauna across all the compared models. This could be caused by the convolutions and pooling layers in the object detection architecture, which gradually reduce the size (and resolution) of the image deeper into the network, making small sized objects harder to detect^51^. Future research could explore backbone network architectures that improve the model’s detection of small-sized objects. Moreover, future research could explore how to extend the FaunD-Fast model to re-use image features extracted from earlier stages of the workflow so as to reduce computation cost, especially for real time megafauna detection while at sea.

Based on the detection results of our trained FaunD-Fast model, we found that the megabenthic fauna abundance in our working area was relatively low. This finding is consistent with previous studies from the CCZ that also reported megafaunal abundances of less than one individual per square meter^52–55^. In terms of regional differences between the two contract areas, a higher abundance was observed in the German area (0.247 ind. m^−2^) compared to the Belgian area (0.200 ind. m^−2^). Similarly, the megafaunal diversity was higher in the German area, with a Shannon diversity index of 2.4 compared to the 1.7 in the Belgian area. Because the German area is located approximately 1050 km east of the Belgian area, the observed high diversity and abundance could be as a result of the east-to-west reduction in the particulate organic carbon flux (POC), as has also been reported in previous studies^56,57^. Also, the difference in abundance between the two contract areas could be explained by availability of food source in the form of sinking organic material through the water column; availability of food is higher in German area because it is on average shallower (−4121 m) than the Belgian area (−4510 m). This relationship between food availability and abundance of megafauna in the Pacific has also been reported in a previous study^58^. Considering the relationship between megabenthic fauna abundance and the seafloor substrate classes from^28^, the Belgian area contained more than 68% of detected megabenthic fauna occupying the large-sized nodules, even though the seabed substrate comprised both large- and densely-distributed nodules. A lower proportion of megabenthic fauna was observed in densely distributed manganese nodules, probably because this substrate class does not allow enough space for soft-sediment dwellers, as was also pointed out in a previous study^59^. On the other hand, analysis in the German area revealed that 57% of megabenthic fauna occurred in seafloor substrates comprising patchy nodules. In addition to being the dominant seafloor class in this area, patchy nodules also provide a natural balance between soft and hard substrates, which would accommodate both hard and soft sediment dwellers, as was also reported in a previous study^59^. In both contract areas, we found that ophiuroids and xenophyophores were the most abundant and diverse morphotypes, and that they occur in association with each other, while occupying both hard and soft bottom substrates. Similar conclusions were also drawn from previous studies^59–61^.

## Conclusion

In conclusion, this study presents an image-based workflow for automatically detecting megabenthic fauna from a sequence of optical underwater images. The workflow relies on the analysis of superpixels to quickly generate a set of weak bounding box annotations, which are then assigned semantic morphotype labels, and used to train a megabenthic fauna detection model (FaunD-Fast). When applied to example seafloor images from the Clarion–Clipperton Zone of the Pacific Ocean, the model achieves good performance, with a mean average precision score of 78.1%. This demonstrates that our approach significantly reduces required effort for generating labeled bounding box annotations, while still achieving performance that is on a par with other state-of-the art object detection models that were trained with labor-intensive manual annotations. Furthermore, our FaunD-Fast model revealed that both the megafaunal abundance and diversity within the German area was greater than in the Belgian area. These findings are also consistent with previous studies that estimated megafaunal abundance in the Pacific using conventional methods. Therefore, we conclude that our workflow provides a convenient, repeatable and accurate machine learning-based approach for automating the detection of megabenthic fauna from huge volumes of underwater image datasets. Because it also reduces required human effort, our approach is easily scalable to seafloor images from other deployments, which makes it quite useful for enabling the efficient monitoring and management of deep-sea marine ecosystems.

## Methods

### Dataset and software

#### Underwater images

The seafloor images used in this study were recorded during twelve video transect deployments in the German and Belgian contract areas for the exploration of polymetallic Mn-nodules in the Clarion–Clipperton Zone of the central Pacific Ocean. The deployments were made on board the German research vessel SONNE during cruise SO268, whose aim was to investigate impacts of nodule mining on the deep seabed environments as part of the JPI-oceans project MiningImpact^62^.

The optical images were recorded from a camera attached to an Ocean Floor Observation Systems (OFOS), which is a towed camera platform that photographs the seabed while at the same time an optical fiber connection to the ship allows online inspection of a video stream from water depths of up to 6000 m^1^. The OFOS was towed by the ship at a speed of ~ 0.5 knots and an altitude between 1 and 4 m above the seafloor. Cumulatively, a track length of 92.5 km across all deployments was investigated at an average water depth of 4280 m. A Canon EOS 5D Mark IV with 24 mm lens was used for recording still images with a frequency of 0.1 Hz, whereas an HD-SDI video camera with a 64° × 64° view angle was used to continuously provide seafloor video footage^62^.

Based on this setup, a total of 40,678 still images were finally used as the example dataset in this study. Each image was georeferenced by matching its acquisition time (UTC) to the ship-based USBL navigation information. All these images are publicly available on PANGAEA^63^.

#### Software development environment

The primary libraries used to implement our megabenthic detection workflow include Python, Scikit-image and the TensorFlow Object Detection API^64,65^. The complete set of the specific python packages, as well as their brief description is provided in the Supplementary Table S3.

Furthermore, we have open-sourced all scripts and modules developed during the implementation of the workflow; these can be accessed online from this repository^66^: https://git.geomar.de/open-source/faund-fast. The repository also contains a detailed documentation that interested users can use as a guide to get started in using our workflow, without the need to re-implement (from scratch) any of the components described in this manuscript.

### Semi-automated generation of weak bounding box annotations

Training state-of-the art object detection models typically requires huge amounts of manually labeled annotations. The required effort can be significantly reduced using weak annotations that can be obtained cheaply. Although these weak annotations may not be directly usable, they can be efficiently refined through post-processing, which then renders them useful for training e.g., an object detection model.

Below, we present a superpixel-based approach for generating weak bounding box annotations. We further describe a following approach for post-processing the weak annotations to remove false positives, and to assign them semantic morphotype labels. Finally, we describe how we used these annotations to train and evaluate a megabenthic fauna detection model.

#### Generation of superpixels

Superpixels were generated using the graph-based segmentation algorithm proposed by^67^. This segmentation algorithm works by first representing an input optical image as a graph, whose nodes are the pixels with edges denoting similarity among connected pixels. Superpixels are then recursively generated from this graph through a series of greedy decisions that group together nodes that have similar characteristics within local neighborhoods. The detailed mathematical formulation of this algorithm can be found in^67^.

**Figure 9:**
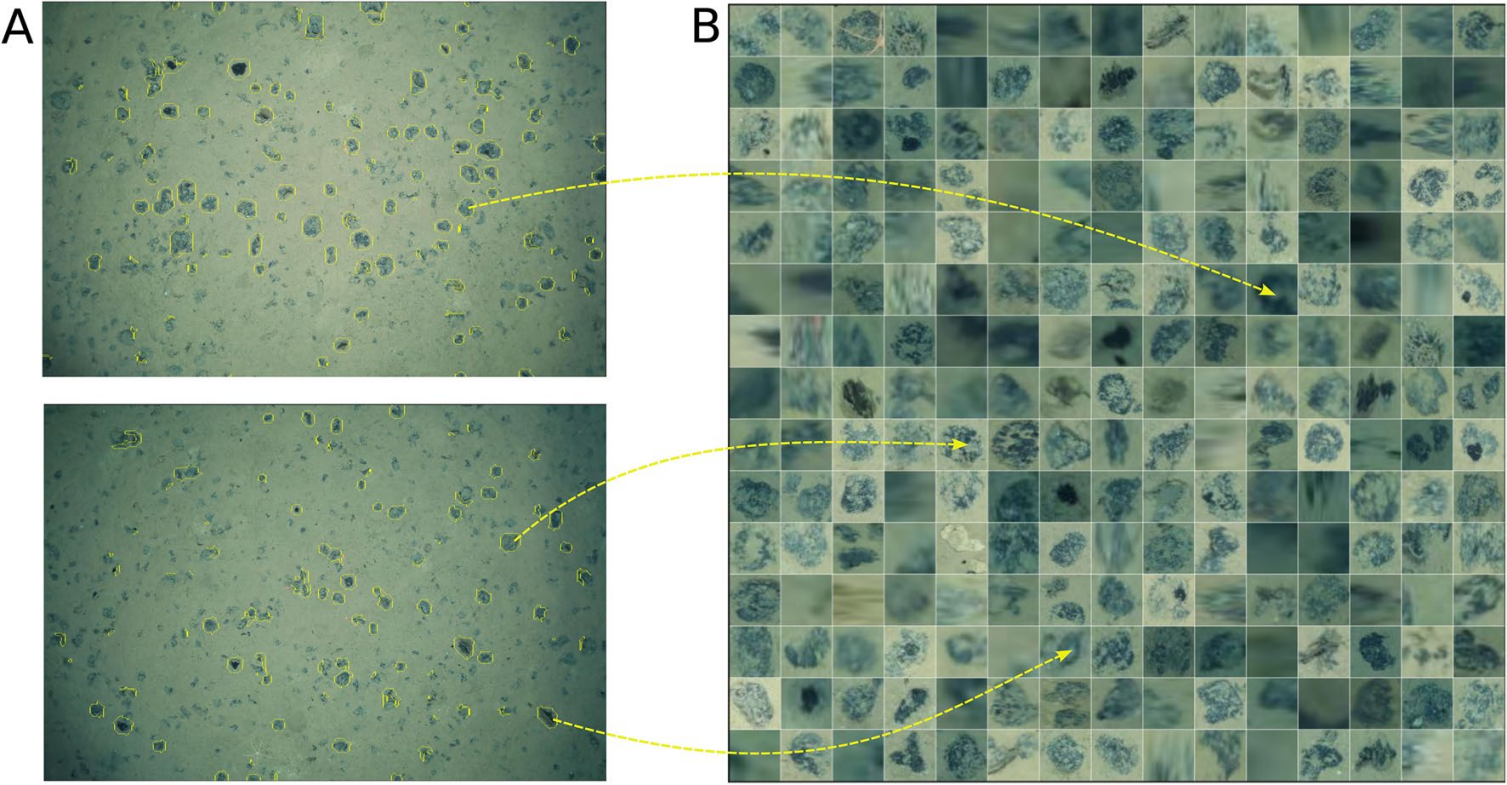
Superpixel generation process. (**A**) Examples of segmented images highlighting boundaries of generated superpixels in yellow, and (**B**) a grid view of a subset of cropped superpixels. Small sized Mn-nodules are not captured by the segmentation because the image was first smoothed with a Gaussian filter to reduce the effect of noisy pixels.

Based on this approach, we generated approximately 250 superpixels for each image; the actual number depends on the visual contents of the respective image e.g., images with densely distributed Manganese (Mn) nodules produced more superpixels than those with plain sediment cover. The generated superpixels were then cropped into square image patches to be used in the subsequent analysis. Figure 9A shows example images with the boundaries of the generated superpixels highlighted, whereas Fig. 9B shows the corresponding cropped image patches. Not visible in Fig. 9A is that the majority of the seafloor sediment is one superpixel, only the smaller isolated superpixel objects are highlighted.

#### Feature extraction from superpixels

We used a convolutional variational autoencoder (VAE) to extract features from the cropped superpixels. This approach was chosen because the features are automatically learnt from the image patches during training, which allowed us to avoid the subjective and non-trivial task of hand-engineering such features^68^. The architecture of the VAE comprises separate encoder and decoder convolutional neural networks that are trained jointly. During each training epoch, the encoder network maps an input superpixel to a vector that represents the parameters of a probability distribution. On the other side, the decoder network takes as input a feature vector that is sampled from this learnt probability distribution, and attempts to reconstruct the input superpixel. In this way, the VAE is trained over several epochs by tuning its parameters to minimize a loss function that consists of both a reconstruction term, as well as a regularization term. Minimizing the reconstruction term reduces the error between the input superpixel and its reconstructed version, whereas the regularization term enforces a Gaussian structure on the learnt probability distribution, which prevents the model from overfitting. Further details on the mathematical formulation of the VAE can be found in^26^.

Based on this approach, we used a trained VAE to extract a 100-dimensional feature vector from each superpixel; this was our chosen optimal number of dimensions that resulted in the lowest value for the loss function. The dimensionality of this feature vector influences the extent to which the encoder network compactly represents the visual information of the input image/patch, and therefore it must be chosen carefully e.g., by performing a parameter search over the space of possible dimensions.

#### Generation of weak annotation from anomalous superpixels

Superpixels were generated from a smaller subset comprising 500 images that were randomly sampled from all the dives; the sampling was done to reduce memory and computational cost when training the anomaly detector model. As expected, the majority of the superpixels generated from these sampled images represent background seafloor, since megabenthic fauna occurs rather infrequently in images of the deep sea^18^. A variational autoencoder was used to extract features from the generated superpixels, and a data matrix was created by stacking the features as row vectors. This data matrix was used to train an instance of the iForest anomaly detection model.

The iForest model is an unsupervised algorithm that uses decision trees to explicitly isolate anomalous superpixels. It works by recursively splitting subsamples of input superpixels based on a randomly selected attribute (column) of the data matrix^27^. The idea is that anomalous superpixels will have unusual values compared to normal superpixels (for a randomly chosen column). As a result, a split e.g., based on a random thresholding of the selected column values will (with high probability) group together the anomalous superpixels in the smaller partition of the split. Thus, by repeatedly performing random splits based on the different columns of the data matrix, it is possible to isolate the superpixels that are anomalous, since they will group together after relatively fewer partitions compared to the normal superpixels.

Thus, after training the iForest model as described above, we applied it (in inference mode) to detect anomalous superpixels from a larger subset comprising images from six dives (it was also necessary to use a subset of images here to reduce computation cost during the CPU-based generation of weak annotations). In addition, the iForest model also assigned an anomaly score to each detected anomalous superpixel, which quantified the extent to which they were deemed anomalous.

#### Post-processing weak annotations to remove false positives

Since the iForest anomaly detection model is fully unsupervised, it inevitably results in a number of false positives that were falsely flagged as anomalous superpixels. While most false positives were already assigned low anomaly scores by the iForest model, the choice of the threshold value of anomaly score that faithfully separates the true and false positives was not obvious. Because of this, we opted to train a binary classifier using the EfficientNet-B0 convolutional neural network architecture^69^. This classifier was then used to automatically separate truly anomalous superpixels from false positives. The selection of training examples for training this binary classifier was relatively straightforward: sorting in descending order of anomaly scores returned most of the truly anomalous superpixels to the top of the list, from which we could quickly select a few relevant positive training examples and vice versa (this selection took approximately an hour).

Thus, we trained the EfficientNet-B0 model using 512 positive examples (of megafauna). The trained model was then used in inference mode to efficiently identify the instances of truly anomalous superpixels (2117), which were then presented to a human annotator as a set of weak annotations.

### Training the megabenthic fauna detector

#### Assigning semantic morphotype labels to weak annotations

So far, the weak annotations only contain bounding box coordinates without corresponding class labels. An expert annotator was invited to manually inspect these weak annotations, and assign appropriate semantic morphotype labels.

To facilitate this labelling, we wrote a custom annotation software that allows the annotator to go through the list of detected superpixels, and view them in the context of the image they were extracted from. A category could then be selected for each superpixel via a shortkey. Furthermore, if the image contained interesting objects that were missed by the anomalous superpixel detector, the software allowed the annotator to draw extra bounding boxes and label them.

Finally, these annotations were exported and split into training (90%) and validation (10%) datasets for subsequent training and evaluation of the Faster R-CNN model. We chose this split criterion because we only had 2118 bounding box annotations; other combinations of train/test split criteria could be adopted if sufficient number of annotations are available.

#### Training the megabenthic fauna detection model

The TensorFlow object detection API^64^ was used for implementing both the training and evaluation pipelines of the megabenthic fauna (object) detection model. From the set of pre-trained object detection models available through this API, we chose the Faster R-CNN model that was paired with a backbone Resnet-101 convolutional neural network architecture^15,70^. We chose this model because it provides a good trade-off between inference speed (76 ms) and mean average precision (37.1 mAP)^64^.

The Faster R-CNN is a two-stage object detection model. The first stage uses the backbone network as a feature extractor that transforms an input image to a set of feature maps, which are used by a region proposal network to generate candidate regions with different scales and aspect ratios that most likely contain an object. The second stage of the model involves two heads: a classification head that classifies the proposed candidate regions by assigning them class probability scores, and a regression head that refines their bounding box coordinates. Since a single object can be detected with multiple overlapping bounding boxes, non-maximum suppression is used in a post-processing step that retains only those boxes which have the highest probability of containing an object^71^. Further details on the mathematical formulation of Faster R-CNN can be found in^15^.

Our training set up comprised a 64-bit computer with 124 Gigabytes of RAM, running an instance of Ubuntu 18.04 LTS as operating system. This computer had 16 CPU cores, as well as a GTX 2080 GPU graphics card with 11 Gigabytes of memory. To speed up the training process, the model was initialized from a detection checkpoint that was pre-trained on the COCO dataset^72^, whereas the backbone network was initialized from the ImageNet classification checkpoint^73^. To reduce the GPU memory requirement given our high-resolution images, we limited the training batch size to only one image per batch. During each epoch of training, the cosine learning rate was used to update the model weights, after warming up for 2000 steps. In total, the model was trained for 100,000 steps, which took approximately 8 h. The configuration file containing settings for the entire set of hyperparameters used during model training is provided in the supplementary material.

Following, we are referring to our proposed megabenthic fauna detection workflow as FaunD-Fast, which is an acronym for Megabenthic Fauna Detection with Faster R-CNN. The acronym encompasses all the components of our proposed workflow as shown in Fig. 1; it is not a renaming of the original Faster R-CNN model architecture.

#### Evaluating the performance of the trained megabenthic fauna detection model

The performance of our trained FaunD-Fast model was evaluated against a validation dataset that was held back during model training. The evaluation followed the standard COCO detection evaluation criteria^72^, which uses metrics such as: (a) precision, which represents the proportion of positive detections that are indeed correct; (b) recall, which represents the proportion of all actual positives that were correctly identified; (c) Intersection-over-Union (IoU), which quantifies the extent of overlap between pairs of bounding boxes.

The intuitive interpretation of these metrics is that for a given IoU threshold, the model with high recall but low precision values will predict very many bounding box detections, most of which are incorrect relative to the ground truths. The opposite is true for a model with high precision but low recall values. Thus, the ideal model is one that has both high precision and high recall, because it predicts many bounding box detections that are also correctly localized and classified.

To evaluate the model performance across all the morphotype classes, the average precision was calculated at single IoU threshold of 0.5 (typically denoted as *AP*_.50_), as well as from an average of 10 different IoU thresholds ranging between 0.5 and 0.95 in incrementing steps of 0.05 (typically denoted as *AP*_.50:0.95_). In addition, both average precision and recall were also calculated for the different coco metric categories as summarized in supplementary Table S4.

Besides calculating the COCO evaluation metrics for our model, we also performed benchmarking with other competing state-of-the art megafauna detection models that were trained and evaluated by Lütjens et al^43^. These models include variants of RetinaNet^74^, CenterMask^75^ and Mask R-CNN^76^. Although the competing models used images from a different cruise (PS118), they are still comparable to our model because all the images were acquired in the same year (2019), using the same camera platform (towed OFOS).

### Estimating megafauna abundance, diversity and spatial distribution

After the performance evaluation, our trained FaunD-Fast model was applied (in inference mode) to the entire underwater image dataset from each of the twelve survey tracks from the two working areas. Because each image (center) is georeferenced, every instance of megafauna was assigned the coordinates of the parent image in which it was detected. Together with the seafloor substrate classification results from^28^, these georeferenced detection results were used to estimate megafaunal diversity using the exponential of Shannon diversity index^77^, as well as their abundance in units of individuals per square meter^78^. In addition to this, the coordinates of the detected megabenthic fauna were used to map their spatial distribution along the camera deployment tracks. The settings for the model parameters described in this “Method” section are provided in the supplementary material.

## Supplementary Information

Supplementary Information.

## Data Availability

The datasets presented in this study can be found online in PANGAEA through the following link https://doi.pangaea.de/10.1594/PANGAEA.935856. Intermediate data generated during the analysis are also provided in the supplementary materials. To request the data used in this study, please contact Prof. Dr. Jens Greinert (jgreinert@geomar.de).
