# Supplementary material for: An automated image-based workflow for detecting megabenthic fauna in optical images with examples from the Clarion–Clipperton Zone
Source: Sci Rep. 2023 May 23;13:8350. doi: 10.1038/s41598-023-35518-5 (PMC10206148; doi:10.1038/s41598-023-35518-5)
Supplement: Supplementary file 1 — Supplementary Information. [file 41598_2023_35518_MOESM1_ESM.zip › supplementary material.pdf]

An automated image-based workflow for detecting megabenthic fauna in optical images with examples from the Clarion-Clipperton Zone

Benson Mbani[1*], Valentin Buck[1] and Jens Greinert[1,2]

[1]DeepSea Monitoring Group, GEOMAR Helmholtz Center for Ocean Research Kiel, Wischhofstraße 1-3, 24148 Kiel, Germany

[2]Institute of Geosciences, Kiel University, Ludewig-Meyn-Str. 10-12, 24118 Kiel, Germany.

*** Correspondence:**
Corresponding Author
bmbani@geomar.de

Keywords: Underwater image analysis, underwater object detection, megafauna detection, image segmentation, superpixels, anomaly detection, ocean floor observation system (OFOS), Clarion-Clipperton Zone (CCZ)

SUPPLEMENTARY FIGURES

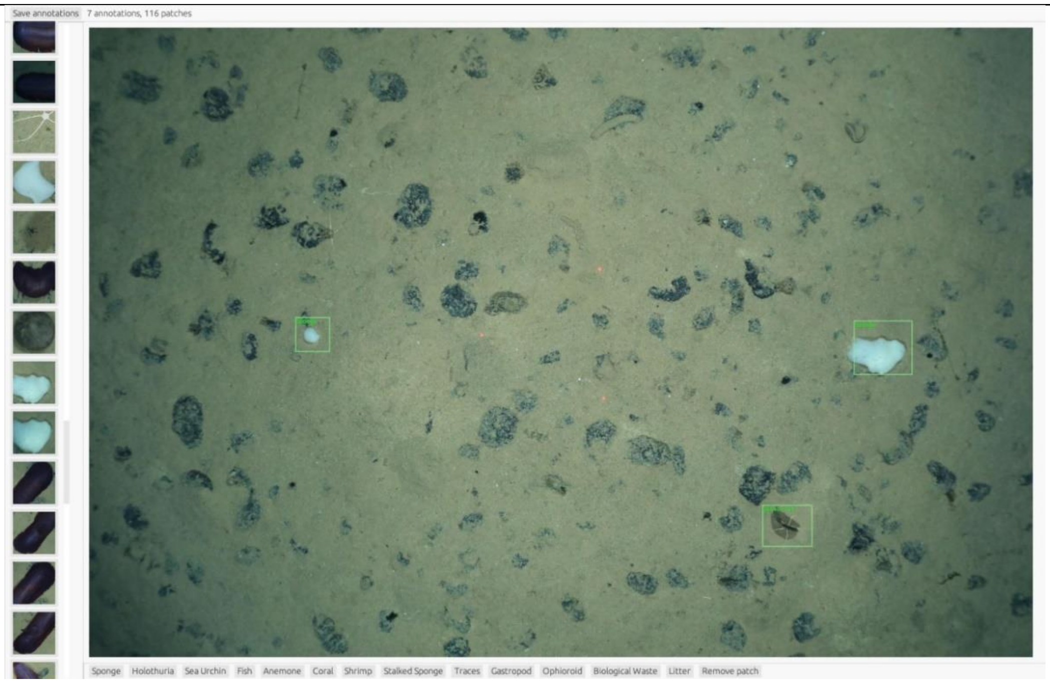

Supplementary Figure S1 - Screenshot of our superpixel annotation software during an active labeling session

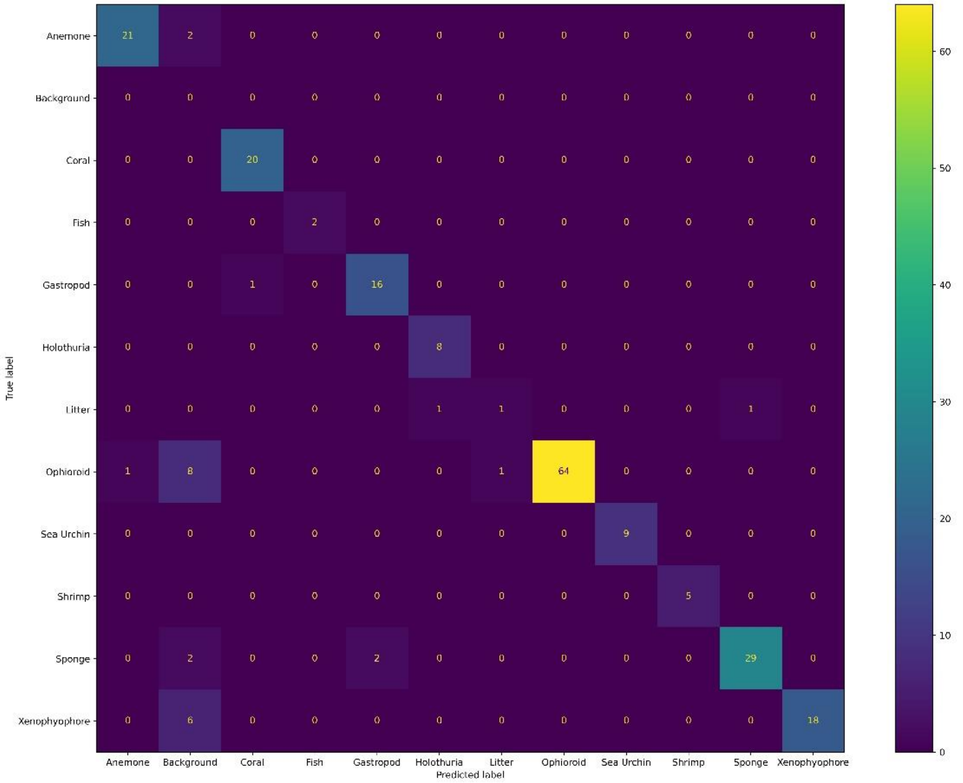

Supplementary Figure S2 – Confusion matrix for semantic evaluation

SUPPLEMENTARY TABLES

Supplementary Table S3 Set of specific python packages (and their description) employed during our software development

Python Library	Usage
**pandas**	Processing navigation text files and managing csv files
**geopandas**	Geotagging images by matching acquisition time vs USBL navigation data
**contextily**	Providing basemaps for cartographic visualization on jupyter notebooks
**tensorflow-gpu**	Transfer learning and image classification for the binary classifier
**cartopy**	Cartographic visualization on jupyter notebooks
**scikit-learn**	Machine learning and dimensionality reduction
**ipython**	Rendering rich text on jupyter notebooks
**shapely**	Computational geometry
**matplotlib**	Visualization and plotting
**imageio**	Reading image datasets into memory
**seaborn**	Visualization and plotting on top of matplotlib
**pillow**	Image processing
**rasterio**	Reading geospatial raster files such as bathymetry, backscatter e.t.c
**scikit-image**	Image processing
**Tensorflow object detection API**	Training and evaluating the object detection model

Supplementary Table S4 Coco metric categories used for performance evaluation to evaluate the performance of our FaunD-Fast object detection model (for detailed descriptions please see https://cocodataset.org/#detection-eval)

**Average Precision (AP)**	
$AP_{.50:.95}$	% AP at IoU=.50:.05:.95
$AP_{.50}$	% AP at IoU=.50
AP_{small}	% AP for small objects: area < 32^2 pixels
AP_{medium}	% AP for medium objects: 32^2 < area < 96^2 pixels
AP_{large}	% AP for large objects: area > 96^2 pixels
**Average Recall (AR)**	
AR_1	% AR given 1 detection per image
AR_{10}	% AR given 10 detections per image
AR_{100}	% AR given 100 detections per image
AR_{small}	% AR for small objects: area < 32^2 pixels
AR_{medium}	% AR for medium objects: 32^2 < area < 96^2 pixels
AR_{large}	% AR for large objects: area > 96^2 pixels

CONFIGURATION FILES

Configuration settings for training the Faster R-CNN model

```
# Faster R-CNN with Resnet-50 (v1)
# Trained on COCO, initialized from Imagenet classification checkpoint

model {
  faster_rcnn {
    num_classes: 11
    image_resizer {
      fixed_shape_resizer {
        width: 1680
        height: 1120
      }
    }
    feature_extractor {
      type: 'faster_rcnn_resnet101_keras'
      batch_norm_trainable: true
    }
    first_stage_anchor_generator {
      grid_anchor_generator {
        scales: [0.25, 0.5, 1.0, 2.0]
        aspect_ratios: [0.5, 1.0, 2.0]
        height_stride: 16
        width_stride: 16
      }
    }
    first_stage_box_predictor_conv_hyperparams {
      op: CONV
      regularizer {
        l2_regularizer {
          weight: 0.0
        }
      }
      initializer {
        truncated_normal_initializer {
          stddev: 0.01
        }
      }
```

```
    }
    first_stage_nms_score_threshold: 0.0
    first_stage_nms_iou_threshold: 0.7
    first_stage_max_proposals: 300
    first_stage_localization_loss_weight: 2.0
    first_stage_objectness_loss_weight: 1.0
    initial_crop_size: 14
    maxpool_kernel_size: 2
    maxpool_stride: 2
    second_stage_box_predictor {
      mask_rcnn_box_predictor {
        use_dropout: true
        dropout_keep_probability: 0.8
        fc_hyperparams {
          op: FC
          regularizer {
            l2_regularizer {
              weight: 0.0
            }
          }
          initializer {
            variance_scaling_initializer {
              factor: 1.0
              uniform: true
              mode: FAN_AVG
            }
          }
        }
        share_box_across_classes: true
      }
    }
    second_stage_post_processing {
      batch_non_max_suppression {
        score_threshold: 0.0
        iou_threshold: 0.6
        max_detections_per_class: 100
        max_total_detections: 300
      }
      score_converter: SOFTMAX
    }
    second_stage_localization_loss_weight: 2.0
    second_stage_classification_loss_weight: 1.0
    use_static_shapes: true
    use_matmul_crop_and_resize: true
    clip_anchors_to_image: true
    use_static_balanced_label_sampler: true
    use_matmul_gather_in_matcher: true
  }
}

train_config: {
  batch_size: 1
  sync_replicas: true
  startup_delay_steps: 0
  replicas_to_aggregate: 8
  num_steps: 100000
  optimizer {
    momentum_optimizer: {
      learning_rate: {
        cosine_decay_learning_rate {
          learning_rate_base: .04
          total_steps: 100000
          warmup_learning_rate: .013333
          warmup_steps: 2000
        }
      }
```

```
      momentum_optimizer_value: 0.9
    }
    use_moving_average: false
  }
  gradient_clipping_by_norm: 10.0
  fine_tune_checkpoint_version: V2
  fine_tune_checkpoint:
"~/fauna_detection_with_tensorflow_object_detection_api/my_model_dir/faster_rcnn_resnet101_v1_102
4x1024_coco17_tpu-8/checkpoint/ckpt-0"
  fine_tune_checkpoint_type: "detection"
  data_augmentation_options {
    random_horizontal_flip {
    }
  }

  data_augmentation_options {
    random_adjust_hue {
    }
  }

  data_augmentation_options {
    random_adjust_contrast {
    }
  }

  data_augmentation_options {
    random_adjust_saturation {
    }
  }


  max_number_of_boxes: 100
  unpad_groundtruth_tensors: false
  use_bfloat16: false  # works only on TPUs
}
train_input_reader: {
  label_map_path:
"~/fauna_detection_with_tensorflow_object_detection_api/data/SO268_label_map.pbtxt"
  tf_record_input_reader {
    input_path: "~/fauna_detection_with_tensorflow_object_detection_api/data/train.tfrecord"
  }
}

eval_config: {
  metrics_set: "coco_detection_metrics"
  use_moving_averages: false
  max_evals: 100
  batch_size: 1;
}

eval_input_reader: {
  label_map_path:
"~/fauna_detection_with_tensorflow_object_detection_api/data/SO268_label_map.pbtxt"
  shuffle: false
  num_epochs: 10
  tf_record_input_reader {
    input_path: "~/fauna_detection_with_tensorflow_object_detection_api/data/validation.tfrecord"
  }
}
```

Configuration settings for training the Faster R-CNN model

```
from pathlib import Path
```

```
DIRECTORY_CONTAINING_IMAGE_DATASET = Path('~/underwater_image_dataset_for_fauna_detection/')

PROJECT_DIRECTORY = Path.cwd().parent

UNSUPERVISED_LEARNING_DIR = PROJECT_DIRECTORY / 'data/unsupervised_outlier_detection/'

DIVE_TO_DETECT_ANOMALIES = 'SO268-1_021-1_OFOS-02' #Specify the folder name

DIVE_PARENT_IMAGES_DIR = DIRECTORY_CONTAINING_IMAGE_DATASET / f'{DIVE_TO_DETECT_ANOMALIES}'

DIVE_WORKING_DIR = UNSUPERVISED_LEARNING_DIR / f'{DIVE_TO_DETECT_ANOMALIES}'

DIVE_SAMPLED_BACKGROUND_IMAGES_DIR = DIVE_WORKING_DIR / 'background_images'

DIVE_PICKLED_ITEMS_DIR = DIVE_WORKING_DIR / 'pickled_items'

DIVE_OUTPUT_DIR = DIVE_WORKING_DIR / 'detection_outputs'

NUMBER_OF_IMAGES_TO_SAMPLE_AS_BACKGROUND = 500

BATCH_SIZE = 32

LATENT_DIMENSION = 100

TRAINING_EPOCHS = 20

CONTAMINATION = 0.01

SUPERVISED_LEARNING_DIR = PROJECT_DIRECTORY / 'data/supervised_fauna_non_fauna_classification/'

DIVE_OUTPUT_DIR_AFTER_BINARY_CLASSIFICATION = SUPERVISED_LEARNING_DIR / 'classification_outputs/'

ANNOTATION_TOOL_DIR = PROJECT_DIRECTORY / 'custom_annotation_tool/'

OBJECT_DETECTION_DIR = PROJECT_DIRECTORY / 'fauna_detection_with_tensorflow_object_detection_api'

EXAMPLE_DIVE = DIVE_TO_DETECT_ANOMALIES

PATH_TO_EXAMPLE_DIVE = UNSUPERVISED_LEARNING_DIR / f'{EXAMPLE_DIVE}'

EXAMPLE_DIRECTORY_WITH_PICKLED_ITEMS = PATH_TO_EXAMPLE_DIVE / 'pickled_items'

MANUSCRIPT_FIGURES_DIRECTORY = PATH_TO_EXAMPLE_DIVE / 'manuscript_figures'

MANUSCRIPT_FIG_SIZE = (12,8)

DETECTION_PATCHES_DIRECTORY = PATH_TO_EXAMPLE_DIVE / 'detection_patches'
```
